# Supplementary material for: Patterns of child mortality in rural area of Burkina Faso: evidence from the Nanoro health and demographic surveillance system (HDSS)
Source: BMC Public Health. 2021 Jul 19;21:1425. doi: 10.1186/s12889-021-11483-4 (PMC8287796; doi:10.1186/s12889-021-11483-4)
Supplement: Supplementary file 1 — Additional file 1: Figure S1. The Nanoro health district is divided to four regions; north, east, south and west of lake. Figure S2. Hazard ratios associated with the probability of mortality of children under-­5. Risk factors reducing the probability of death have hazard ratios lower than 1 to the left of the vertical dashed line. Hazard ratios (yellow points), 95% confidence intervals (horizontal lines) and the p values are given. The variables with the yellow points on the vertical dashed line represent the reference groups. Figure S3. Mortality per 1000 live births per village for children under­-5. Each circle represents a village and the size of circle represents the number of live births. The lighter color indicates lower mortality rate and darker color indicates a higher mortality rate. Figure S4. Travel time of children under-­5, living in the Nanoro health district, from an inpatient health facility. Each circle represents an individual. Figure S5. Mortality per 1,000 live births for children under­-5 versus subgroups of distance, travel time and walking travel time to inpatient health facilities. Circle size represents the number of births within each subgroup. Figure S6. Mortality per 1,000 live births for children under­-5 versus subgroups of distance, travel time and walking travel time to outpatient health facilities.Circle size represents the number of births within each subgroup. Figure S7. Mortality per 1000 live births for each event month. Figure S8. Mortality count by age group. Figure S9. In-­ and out-­migration count by month. Table S1. Results of unadjusted Cox regression models for under­5 mortality. Hazard Ratios are presented with 95% confidence intervals in parentheses. Table S2. Results of unadjusted cox regression models for under­-5 mortality per 1000 live births. Each risk factor was fitted to the model as a continuous variable. Hazard Ratios are presented with 95% confidence intervals in parentheses. [file 12889_2021_11483_MOESM1_ESM.pdf]

Supplementary Material:

Spatial patterns of child mortality in Nanoro HDSS site, Burkina Faso

Navideh Noori[1*¥], Karim Derra[2¥], Innocent Valea[2,3], Assaf P. Oron[1], Palwende Romuald Boua[2], Aminata Welgo[2], Toussaint Rouamba[2], Athanase M. Somé[2], Eli Rouamba[2], Edward Wenger[1], Hermann Sorgho[2], Halidou Tinto[2,3], and Andre Lin Ouédraogo[1]

[1]Institute for Disease Modeling, Global Health Division, Bill & Melinda Gates Foundation, 500 5th Ave N, Seattle, WA, 98109, USA.

[2]Institut de Recherche en Sciences de la Sante (IRSS)/Clinical Research Unit of Nanoro (CRUN), Nanoro, Burkina Faso

[3]Laboratory of Parasitology and Entomology, Centre Muraz, Bobo-Dioulasso, Burkina Faso

[*]Corresponding author

[¥]Equally contributed

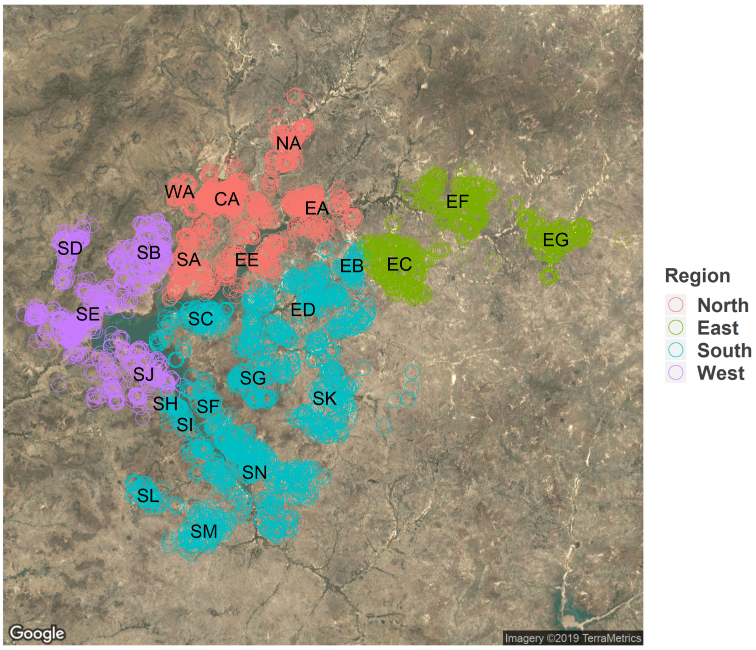

Figure S 1. The Nanoro health district is divided to four regions; north, east, south and west of lake.

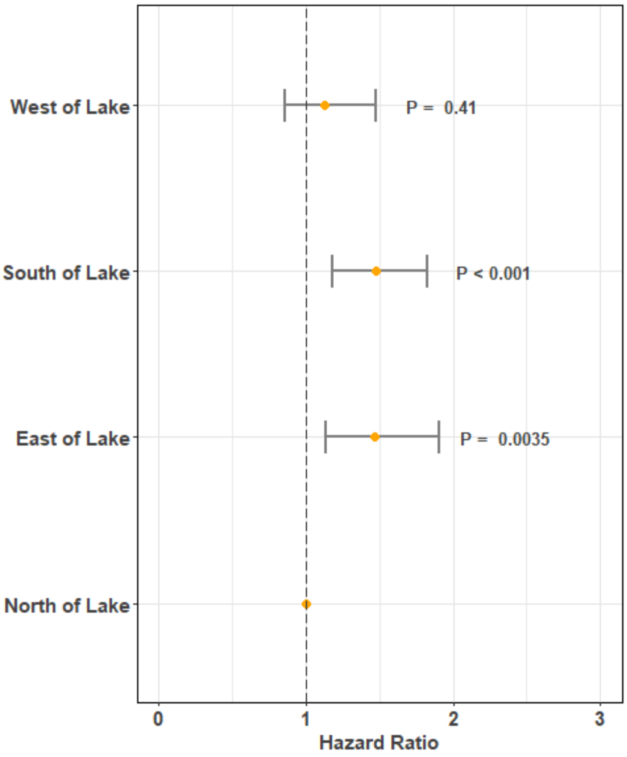

Figure S 2. Hazard ratios associated with the probability of mortality of children under-5. Risk factors reducing the probability of death have hazard ratios lower than 1 to the left of the vertical dashed line. Hazard ratios (yellow points), 95% confidence intervals (horizontal lines) and the p values are given. The variables with the yellow points on the vertical dashed line represent the reference groups.

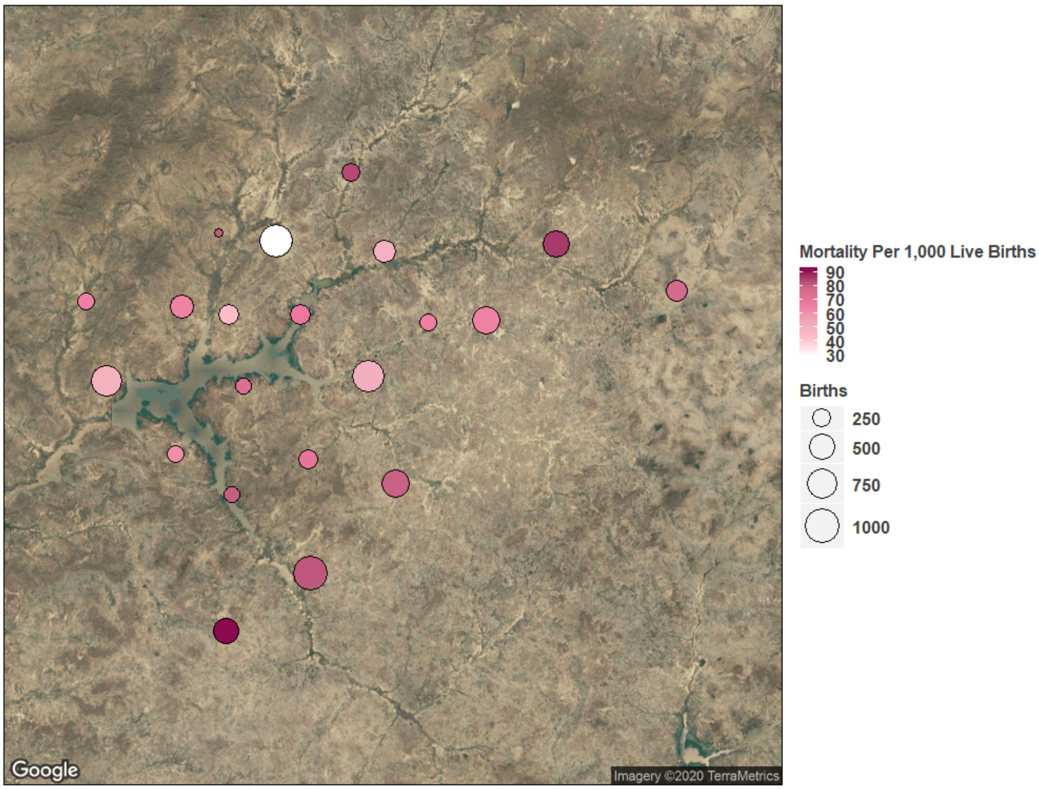

Figure S 3. Mortality per 1,000 live births per village for children under-5. Each circle represents a village and the size of circle represents the number of live births. The lighter color indicates lower mortality rate and darker color indicates a higher mortality rate.

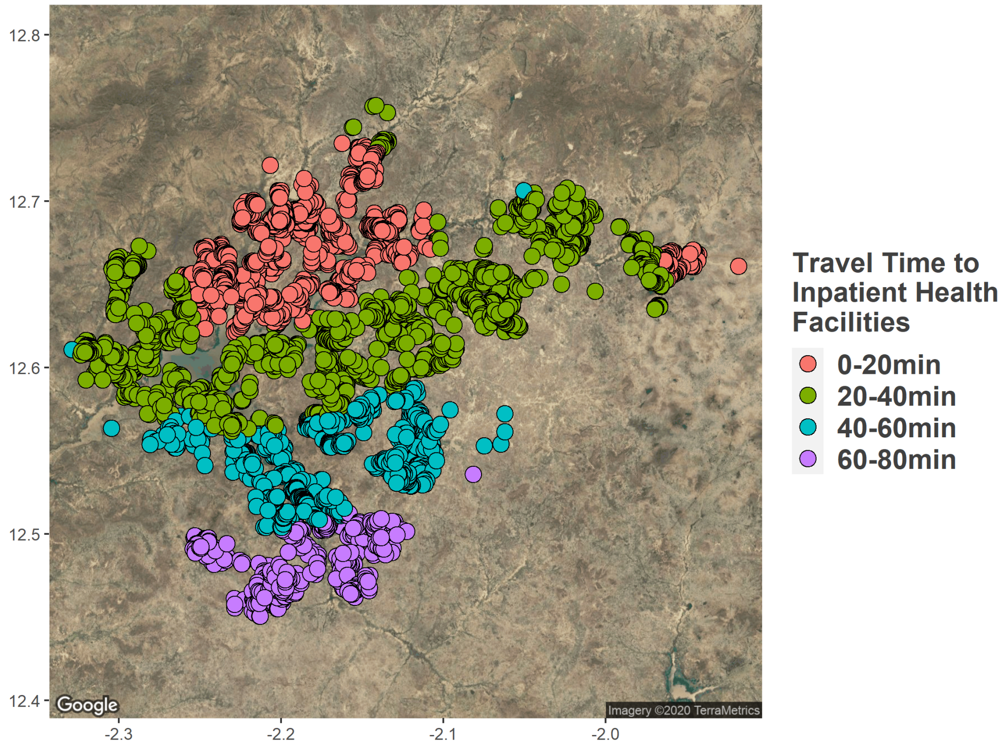

Figure S 4. Travel time of children under-5, living in the Nanoro health district, from an inpatient health facility. Each circle represents an individual.

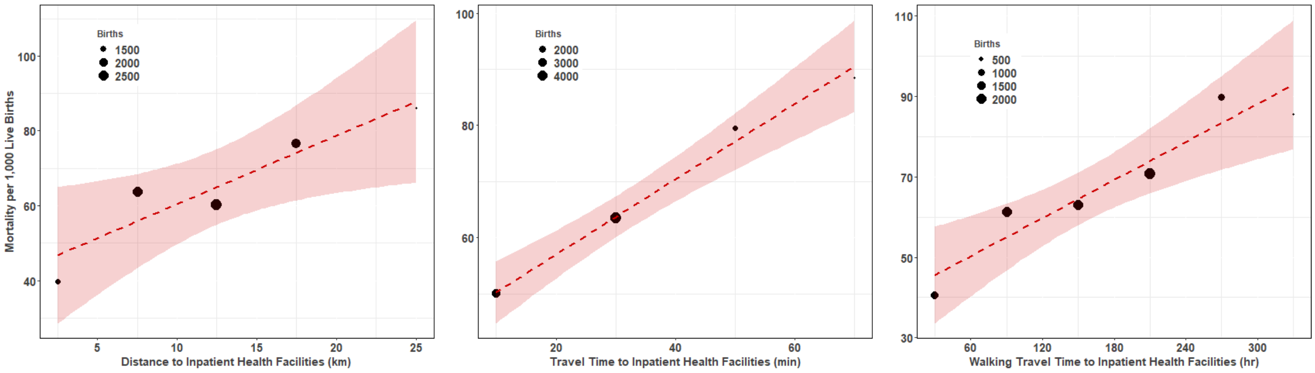

Figure S 5. Mortality per 1,000 live births for children under-5 versus subgroups of distance, travel time and walking travel time to inpatient health facilities. Circle size represents the number of births within each subgroup.

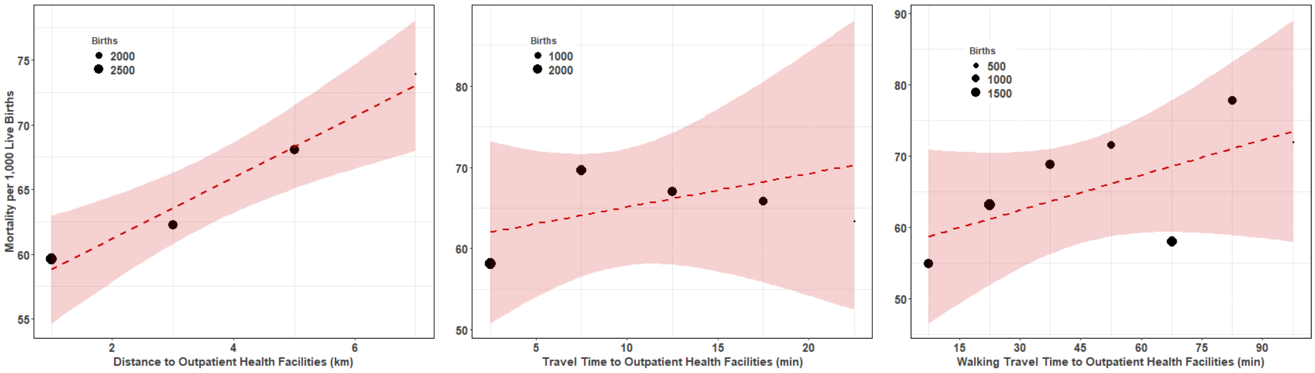

Figure S 6. Mortality per 1,000 live births for children under-5 versus subgroups of distance, travel time and walking travel time to outpatient health facilities.Circle size represents the number of births within each subgroup.

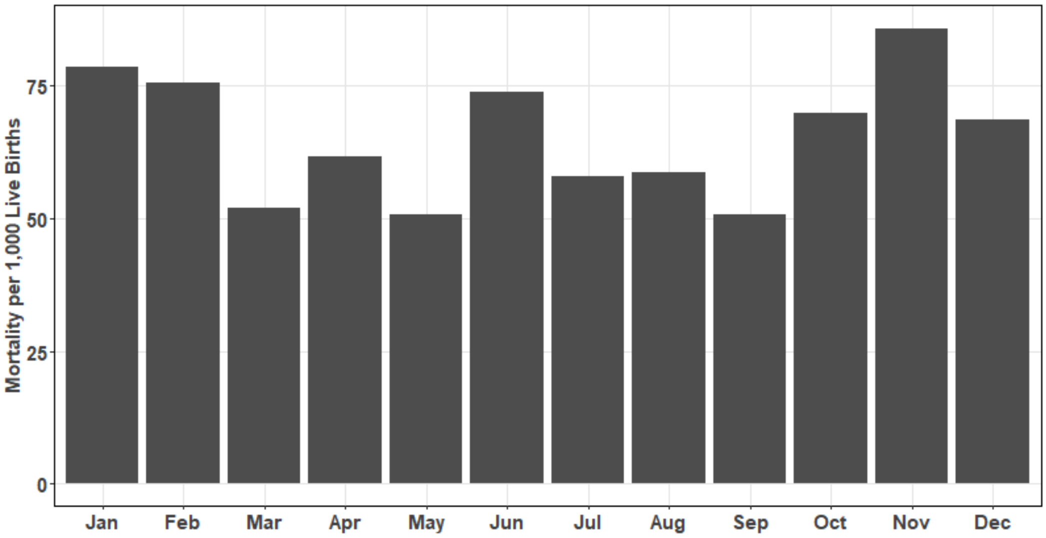

Figure S 7. Mortality per 1,000 live births for each event month.

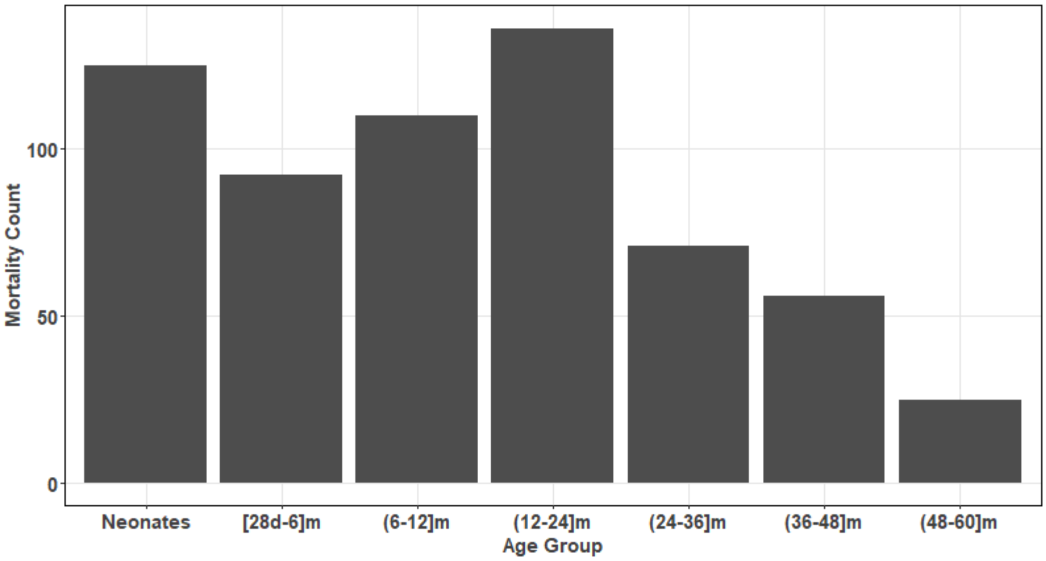

Figure S 8. Mortality count by age group.

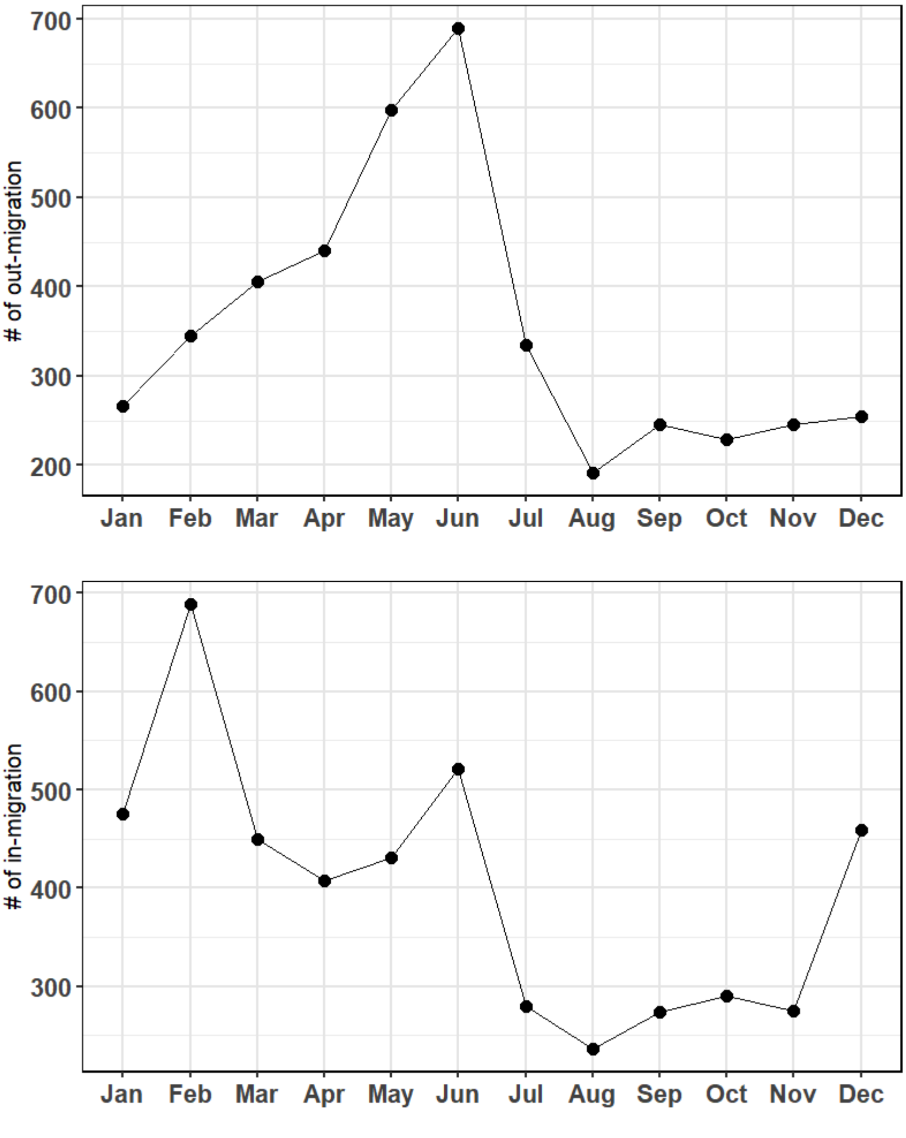

Figure S 9. In- and out-migration count by month.

Table S 1. Results of unadjusted Cox regression models for under-5 mortality. Hazard Ratios are presented with 95% confidence intervals in parentheses.

Parameters	Mortality per 1,000 live births	Hazard ratio	P-value
**Distance to inpatient health facilities (km)**			
$[0-5)$	39.69	-	-
$[5-10)$	63.70	1.48 (1.02,2.24)	0.045
$[10-15)$	60.19	1.42 (0.94,2.22)	0.11
$[15-20)$	76.52	1.77 (1.16,2.69)	0.008
$20+$	86.05	2.04 (1.34,3.09)	< 0.001
**Walking travel time to inpatient health facilities (hr)**			
$[0-1)$	40.39	-	-
$[1-2)$	61.31	1.39 (0.94, 2.07)	0.09
$[2-3)$	62.98	1.45 (0.95, 2.21)	0.08
$[3-4)$	70.77	1.63 (1.05, 2.53)	0.03
$[4-5)$	89.92	2.05 (1.35, 3.108)	< 0.001
$5+$	85.5	2.08 (1.18, 3.66)	0.01
**Distance to outpatient health facilities (km)**			
$[0-2)$	59.6	-	-
$[2-4)$	68.07	1.13 (0.85, 1.5)	0.43
$[4-6)$	62.27	1.03 (0.76, 1.4)	0.85
$6+$	73.93	1.19 (0.84, 1.72)	0.32
**Walking travel time to outpatient health facilities (min)**			
$[0-15)$	54.89	-	-
$[15-30)$	63.1	1.14 (0.68,1.92)	0.62
$[30-45)$	68.77	1.26 (0.79,2.02)	0.34
$[45-60)$	71.5	1.24 (0.73,2.10)	0.41
$[60-75)$	58.09	1.05 (0.64,1.73)	0.85
$[75-90)$	77.8	1.37 (0.79,2.35)	0.24
$90+$	71.9	1.31 (0.72,2.37)	0.38
**Gender**			
Male	66.87	-	-
Female	62.83	0.94 (0.80,1.1)	0.43
**Religion**			
Animist	71.71	-	-
Catholic	54.58	0.85 (0.67, 1.083)	0.19
Muslim	66.14	1.03 (0.83, 1.29)	0.75
Protestant	51.65	0.74 (0.52,1.05)	0.1
Unknown	155.04	1.82 (1.24, 2.65)	0.002
**Ethnicity**			
Mossi	63.61	-	-
Peulh	43.67	0.73 (0.38, 1.41)	0.35
Gourounsi	60	0.93 (0.77,1.12)	0.43
Unknown	137.4	1.68 (1.31,2.16)	< 0.001
**Maternal education status***			
No Education	43.79	-	-
Some Education	20.07	0.48 (0.27,0.84)	0.01
**Multiple birth status***			
Singleton	39.20	-	-
Twin/Triplet/Quadruplet	97.11	2.59 (1.72,3.91)	$<$ 0.001

* Children born after the HDSS establishment.

Table S 2. Results of unadjusted cox regression models for under-5 mortality per 1,000 live births. Each risk factor was fitted to the model as a continuous variable. Hazard Ratios are presented with 95% confidence intervals in parentheses.

Parameters	Children Under-5	
	Hazard Ratio	P-value
Event month	1.05 (1.01,1.8)	0.009
Age (month)	0.97 (0.96,0.98)	< 0.001
Distance to inpatient health facilities (km)	1.03 (1.015,1.048)	< 0.001
Travel time to inpatient health facilities (min)	1.01 (1.004,1.015)	< 0.001
Walking travel time to inpatient health facilities (min)	1.002 (1.001,1.004)	< 0.001
Distance to outpatient health facilities (km)	1.022 (0.96,1.089)	0.46
Travel time to outpatient health facilities (min)	1.008 (0.985,1.031)	0.51
Walking travel time to outpatient health facilities (min)	1.002 (0.997,1.007)	0.40
